# Pseudo-Bootstrap Network Analysis—an Application in Functional Connectivity Fingerprinting

**DOI:** 10.3389/fnhum.2017.00351

**Published:** 2017-07-13

**Authors:** Hu Cheng, Ao Li, Andrea A. Koenigsberger, Chunfeng Huang, Yang Wang, Jinhua Sheng, Sharlene D. Newman

**Affiliations:** ^1^Department of Psychological and Brain Sciences, Indiana University, Bloomington IN, United States; ^2^Department of Statistics, Indiana University, Bloomington IN, United States; ^3^Department of Radiology, Medical College of Wisconsin, Milwaukee WI, United States; ^4^College of Computer Science, Hangzhou Dianzi University, Hangzhou Zhejiang, China

**Keywords:** random parcellation, pseudo-bootstrap, network analysis, functional connectivity fingerprint, connectomes, intra-class correlation coefficient

## Abstract

Brain parcellation divides the brain’s spatial domain into small regions, which are represented by nodes within the network analysis framework. While template-based parcellations are widely used, the parcels on the template do not necessarily match individual’s functional nodes. A new method is developed to overcome the inconsistent network analysis results by by-passing the difficulties of parcellating the brain into functionally meaningful areas. First, roughly equal-sized parcellations are obtained. Second, these random parcellations are applied to individual subjects multiple times and a pseudo-bootstrap (PBS) of the network is obtained for statistical inferences. It was found that the variation of mean global network metrics from PBS sampling is smaller compared with inter-subject variation or within-subject variation between two diffusion MRI scans. Using the mean global network metrics from PBS sampling, the intra-class correlation is always higher than the average obtained from using a single random parcellation. As one application, the PBS method was tested on the Human Connectome Project resting state dataset to identify individuals across scan sessions based on the mean functional connectivity (FC)—a trivial network property that has little information about the connectivity between nodes. An accuracy rate of ∼90% was achieved by simply finding the maximum correlation of mean FC of PBS samples between two scan sessions.

## Introduction

Network analysis provides a complete new avenue in exploring the function and structure of the brain from a network perspective (Brodmann, 1909). A network comprises nodes and edges. One of the biggest challenge of network analysis in neuroimaging is defining the nodes (de Reus and van den Heuvel, 2013; Stanley et al., 2013). The most natural way to define nodes would be to represent individual neurons as nodes. However, even a single voxel in the brain image contains millions of neurons. A parcellation in the microscopic level is unrealistic for MRI-based whole brain imaging. Historically, people have attempted to divide the brain into different regions with similar anatomical or functional features (Brodmann, 1909; Tzourio-Mazoyer et al., 2002; Fischl et al., 2004). Lately, the Human Connectome Project (HCP) has become a driving force for brain parcellation (Craddock et al., 2012; Shen et al., 2013; Glasser et al., 2016; Gordon et al., 2016). Brain parcellation divides the brain’s spatial domain into small regions, which serve as nodes for network analysis. In general, a brain parcel is a region that has greater commonality of features within the parcel than with neighboring parcels. Many parcellation schemes have been developed in the last decades using anatomical landmarks, functional connectivity (FC), and multimodal approaches. For instance, Freesurfer generates a cortical atlas based on the curvature values of gyral and sulcal regions derived from a T1-weighted image (Fischl et al., 2004; Desikan et al., 2006); the AAL parcellation draws 116 regions based on the brain sulci of a MNI MRI Single-Subject (Tzourio-Mazoyer et al., 2002). Craddock et al. (2012) developed an algorithm to parcellate the whole brain into spatially coherent regions of homogeneous FC. A similar idea was further developed by incorporating graph theory and groupwise clustering of a group of subjects (Shen et al., 2013). Lately, multi-modal MRI images from the HCP have been used for parcellation (Glasser et al., 2016). The images from 210 healthy young adults were precisely aligned. One hundred and eighty areas per hemisphere were obtained from group averaging of multi-modal information in cortical architecture, task activation, resting state FC, and/or topography. Although more sophisticated algorithms and novel approaches have been incorporated into brain parcellation (Glasser et al., 2016; Gordon et al., 2016), there is no consensus as to what is the “perfect parcellation” and limitations set by the data make the problem even more challenging.

An alternative to feature-based parcellation is random parcellation (Fornito et al., 2010; Zalesky et al., 2010; Echtermeyer et al., 2011; de Reus and van den Heuvel, 2013). Instead of parcellation based on structural or functional features of the brain, random parcellation generates parcels with little constraint other than contiguity in space and similar size. The fewer constraints have the advantage of enabling the creation of parcellations with an arbitrary number of nodes, which is desirable to carry out multi-scale network analysis (Fornito et al., 2010). Another advantage of random parcellation is that given a certain number of nodes, there are many ways to parcellate the brain, a feature that allows us to study robustness of network-based analysis.

When comparing networks between subjects, a widely used approach is to obtain a parcellation template and apply it to all subjects. The parcellation template can be derived with any of the above schemes including random parcellation. The widely used parcellation templates include AAL (Tzourio-Mazoyer et al., 2002), Shen atlas (Shen et al., 2013), Craddock atlas (Craddock et al., 2012), etc. Template-based parcellation provides a common framework in comparing networks from different subjects as it offers a one-to-one map between node-level measures and, it allows direct comparison of global-network measures, given that the magnitude of most network metrics are highly dependent on network size (van Wijk et al., 2010; Zalesky et al., 2010). However, challenges remain in comparing networks between subjects because of the variability across individuals and internal heterogeneity in multiple levels such as columnar organization and subcellular/cellular structures (Glasser et al., 2016). For template-based parcellations, the parcels on the template do not necessarily match individual’s functional nodes, which are supposed to be homogeneous in performing functional tasks. In addition, different functional task might evoke different brain regions. In other words, the nodes should not be considered fixed at the macroscopic level (Gordon et al., 2016). The majority of the parcellation scheme can be regarded as a coarse sampling of the nodes with some constraints such as that the voxels are contiguous and coherent in time course.

Taking a slightly different view, parcellation is a sampling of millions of neurons with some constraints. Then we need to take into account the ambiguity of this sampling at the microscopic level. We propose to use multiple random parcellation as a pseudo-bootstrap (PBS) sampling scheme. For each subject, a set of networks can be obtained from multiple random parcellations, which is essentially a resample of the same data, a technique often used in statistics (Efron and Tibshirani, 1994). Of course, there must be some constraints on the set of randomly generated parcellations that conform the sampling set, such as number of nodes, node size, etc. These constraints are implemented through the appropriate choice of algorithm that generates the parcellations. Therefore, this method is considered a PBS approach. An important benefit of this method is that it gives the probability of parcellation-related distribution of global network metrics. A striking difference of this method from conventional bootstrap method is that the number of samples is much smaller than the actual data points. Given that there is no golden standard for brain parcellation at the macroscopic level, PBS sampling can be an appealing approach.

A requirement of the PBS network analysis method is to consider random parcellations with roughly equal parcel size (de Reus and van den Heuvel, 2013). The roughly equal parcel size ensures the consistency of the multiple sampling so that the variation of network properties comes solely from resampling rather than size differences. It is challenging to generate equal-sized parcels because of the irregularity of the cortical surface. Previous random parcellation algorithms achieved the inter-quartile range to median ratio of 0.77 (Fornito et al., 2010) and 0.52 (Echtermeyer et al., 2011), which is not satisfactory for this purpose. We have developed a new algorithm to improve the homogeneity of parcel size by taking account of the geodesic distance between voxels and variation of voxel density across the cortical area.

PBS network analysis using 400 random parcellation generated from our new algorithm was exerted on the structural network derived from diffusion MRI (dMRI). Basic statistical properties were evaluated on some global network metrics. The intra-class correlation coefficients (ICCs) were computed accordingly and compared with template-based parcellations. As one application, the PBS network analysis was employed on the HCP resting state dataset to identify individuals across scan sessions based on the mean FC (Finn et al., 2015).

## Materials and Methods

### Random Parcellation

Segmentation was performed on a T1-weighted anatomical image from the HCP with the FSL tool FAST (Zhang et al., 2001; Smith et al., 2004). The gray matter mask was obtained by setting the threshold of 0.5 on the probabilistic gray matter map. Then random parcellation was performed on the gray matter mask based on the algorithm described in (Zalesky et al., 2010). The algorithm produces random parcellations by growing voxel neighborhoods around a set of randomly selected voxel-seeds. After randomly placing the first voxel-seed, all subsequent seeds are placed in a deterministic manner by the distance measure before growing neighborhoods iteratively. However, the distance of the seeds in the original algorithm was computed based on Euclidean distance. Because the cortical surface is very irregular, using Euclidean distance as a measure to ensure that seeds are evenly placed throughout the cortical surface results in large parcel-size variation.

Here, we introduce a geodesic distance *G*(*i,j*), which is the topological shortest path between voxels *i* and *j*, where such path is restricted to traversing voxels within the gray matter surface. The computation of geodesic distance can be converted to a problem of calculating the path length of a weighted network, of which each node is represented by a gray matter voxel and is only connected to its spatially contiguous neighbors. The connection weights between adjacent voxels are defined as follows: w_ij_ = 1 if voxels *i* and *j* share a face; 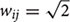 if *i* and *j* share one side; 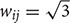 if *i* and *j* share a vertex. Hence, it is straightforward to obtain the geodesic distance *G*(*i,j*) between any voxels by simply calculating the corresponding path lengths between all node pairs (voxels) of the network.

To minimize the variation in parcel size, we further weighted the geodesic distance by local density of the voxels because higher local density means less hindrance in growing the volume. Thus, the distance in *D*(*i,j*) is finally defined as

(1) $$D\left(\mathrm{i,j}\right)=\frac{\mathrm{2G}\left(\mathrm{i,j}\right)}{L(i)+L\left(j\right)}$$

where *L*(*i*) is the sum of shortest-path lengths between voxel *i* and its *M* nearest neighbors, and *M* is the expected number of voxels within a parcel, given a specified number of parcels *N*.

The parcellation algorithm was implemented in Matlab (The Mathworks, Inc., Natick, MA, United States). To evaluate the homogeneity of parcel size and compare with previous random parcellation results, the algorithm was tested a large range of number of nodes *N* = 125, 250, 500, and 1000. Two hundred repetitions were run for each value of *N*, except for *N* = 250 nodes, where 600 repetitions were run.

In addition, 400 random parcellation with 278 ROIs on the MNI template were obtained for the network analysis in Sections “Structural Network” and “Finger Printing of Functional Network.” The parcellations were obtained from the same cortical region of the Shen atlas fconn_atlas_150_2mm.

### Structural Network

Forty-six subjects received two dMRI scans with one week apart. The dMRI data were acquired on a 3.0 T TIM Trio scanner using a 12-channel head coil. The imaging parameters were as following: TR/TE = 8300/77 ms; 68 transversal slices with isotropic 2 mm resolution; 48 diffusion directions with gradients *b* = 1000 s/mm^2^, and eight samplings at *b* = 0. A high resolution T1-weighted image was acquired with the MP-RAGE pulse sequence (1 mm isotropic resolution, TR/TE = 2300/2.91 ms, TI = 900 ms, FA = 9).

The dMRI data were processed with FSL and tractography was computed using the FACT algorithm (Mori et al., 1999) using Diffusion Toolkit^1^ as described previously in more detail (Cheng et al., 2012).

The parcellation on the MNI template was warped to the diffusion space with the help of the T1-weighted anatomical image. As a result, the parcellation and the tractography were coregistered. The structural network was constructed by defining the weight of edges as the number the fibers connecting a pair of nodes normalized by the mean volume of the two ROIs and the mean fiber length between the two ROIs (Hagmann et al., 2007), as described in Eq. 2:

(2) $$w_{ij}=\frac{2}{n_{i}+n_{j}}\underset{m}{\Sigma }\frac{1}{L_{ij}^{m}}$$

where *n_i_* denotes the number of voxels in ROI*_i_*, 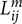 denotes the length of the *m*th fiber between ROI*_i_* and ROI*_j_*. To reduce the effect of spurious fibers, a threshold of 10 fibers is set that two nodes are not connected if the number of fibers between them is smaller than 10. Four hundred networks were obtained from random parcellation along with one network constructed using the template-based parcellation. Six global network metrics were computed including the average degree, mean strength, mean clustering coefficient, global efficiency, modularity, and mean diversity. We computed the variation of global network metrics associated with the set of random parcellations, dMRI scans, and subjects. The variation of global network metrics from parcellation was simply the standard deviation of the global metrics across 400 networks generated from the random parcellations. The between scan variation was computed as

(3) $$\sigma_{BS}=\sqrt{\frac{1}{M}\overset{M}{\underset{i=1}{\Sigma }}({\overset{-}{G}}_{1i}-{\overset{-}{G}}_{2j}{)}^{2}}$$

where *M* is the number of subjects, and 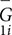 is the mean global metric of subject *i* from scan 1. The variation from inter-subject difference was calculated as the standard deviation of the mean global metrics across all subjects at scan 1.

We also used the ICC (Shrout and Fleiss, 1979) as an index to compare PBS parcellation and template-based parcellation. The ICC is a measure of how much between-subject variation contributes to the total variance. For PBS analysis, there are two ways to compute the ICC. The first method uses the mean value of the global metrics for each subject/measurement; the second method computes the ICC of each parcellation and then calculate the mean ICC value. A tailed *t*-test was performed to compare PBS using the mean and template-based parcellation using one random parcellation. The ICC was also computed for the Shen atlas.

### Finger Printing of Functional Network

Resting state functional data from 87 subjects were downloaded from the data release of the HCP (Q1 through Q3). Each subject has two sessions of resting state fMRI scans: REST1 and REST2, which are one day apart. The dataset have been preprocessed and normalized to the MNI template via non-linear transformation. Using the random parcellation obtained in Section “Random Parcellation” that shared the same cortical space as the Shen atlas, FC was computed as the Pearson pair-wise correlation between the time series of the nodes after regressing motion parameters as well as signal from the white matter and CSF, resulting a 278 × 278 matrix for each parcellation. A template-based FC network constructed from the Shen atlas was also obtained. The functional finger print predicts a subject *i* in REST1 with ID 1*i* to be one of the subjects in REST2 with ID 2*k* if the similarity between the FC of ID_1_*_i_* and ID_2_*_j_* was maximized among all subjects in REST2,

(4) $${ID}_{1i}={ID}_{2k},\;\;where\,\;\;\;k\,\;=\arg\;\;\underset{k}{\max}\;similarity\,\;\;\left({ID}_{1i},{ID}_{2k}\right)$$

The accuracy for the subject *i* in REST1 was calculated as 1 if ID_1_*_i_* = ID_2_*_i_* and 0 otherwise. Identifying individuals of REST2 from REST1 is vice versa. In the work by Finn et al. (2015), a correlation of the template-based FC matrices was used as the measure of similarity. We propose a new measure of similarity to take advantage of the PBS parcellation. Each subject has 400 such FC matrices per session, and the mean of the FC forms a vector of 400 elements. This vector was named as the mean FC vector (mFCV). To use the FC as a fingerprint to identify subjects across resting state fMRI scans, we define the similarity as the cross-correlation of the mFCV between subjects. As a comparison, we also used the cross-correlation as similarity to calculate fingerprinting accuracy with Shen atlas and single parcellation of PBS sampling.

## Results

### Random Parcellation

**FIGURE 1:**
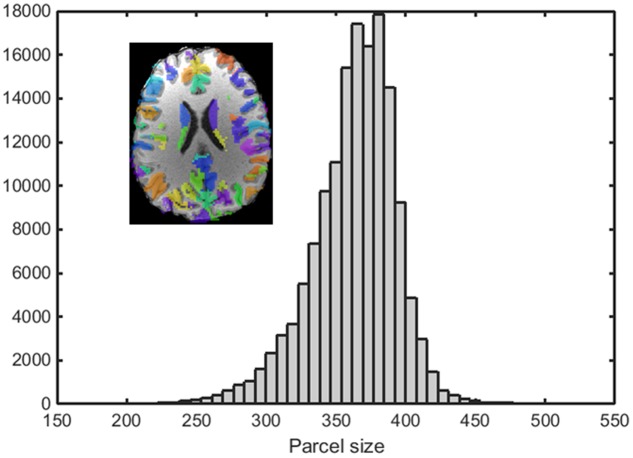
An example of a random parcellation of 250 nodes generated with our algorithm (inset) and the corresponding parcel-size distribution.

An example of a 250-node random parcellation generated with our algorithm and the corresponding parcel-size distribution are shown in **Figure 1**. The ratio of standard deviation to the mean parcel size is 8.4%. Across all 600 trials, 95% of the parcel-sizes are between 291 voxels and 413 voxels, and 99% of the parcel-sizes are between 257 voxels and 434 voxels. If we define the normalized maximum variation (NMV) as the biggest difference in size of a parcellation, divided by the smallest parcel size, the mean value is 79.3% across 600 repetitions, with the smallest NMV of 38.8 and 87.7% of the trials resulting in NMV < 100%. **Table 1** summarizes some features of the distributions obtained for different values of *N*. The inter-quartile range to median ratio is 10% for 500 parcels and 12% for 1000 parcels, much smaller compared to previous reported values of random parcellation with 0.77 for 890 parcels (Fornito et al., 2010) and 0.52 for 813 parcels (Echtermeyer et al., 2011).

**Table 1:** Characteristics of the random parcellation generated with our algorithm.

Number of	Parcel size	Standard deviation	Inter-quartile range
nodes	(voxels)	to mean ratio (%)	to median ratio
125	725.7 ± 65.1	8.97	0.11
250	362.9 ± 30.6	8.43	0.10
500	181.4 ± 15.7	8.68	0.10
1000	90.7 ± 8.4	9.28	0.12

### Structural Network

#### Statistical Distribution of the Global Metrics

**FIGURE 2:**
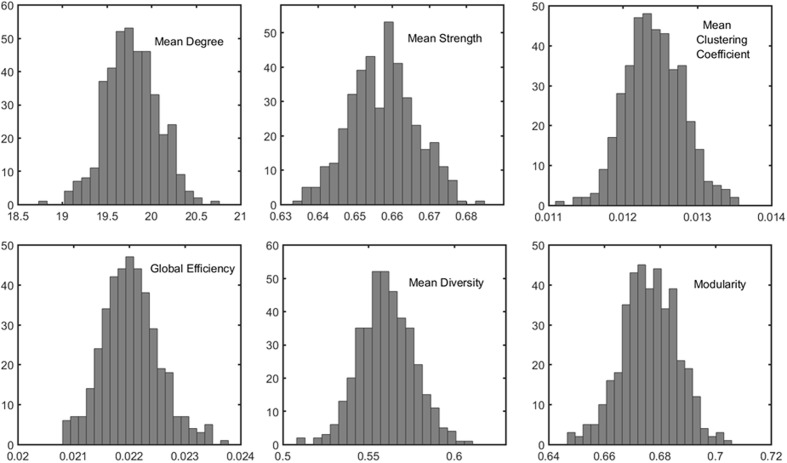
Example of the distribution of six global metrics from the PBS network analysis of one subject. The results were from 400 random parcellation networks.

The distributions of some network metrics from 400 trials of the random parcellations with *N* = 278 are shown in **Figure 2**. A Lilliefors test showed that the distributions are not significantly different from a normal distribution. **Table 2** listed variations of six global network metrics associated with parcellation, along with those between MRI scans, and those induced by inter-subject variability. The parcellation-related variations are much smaller for five of the six global metrics compared with within subject differences and between subject differences.

**Table 2:** Variation of six global network metrics associated with parcellation, between MRI scans, and between subjects.

	Parcellation	Between scans	Between subjects
Degree	0.274	0.934	0.975
Strength	9.12e-3	36.7e-3	42.3e-3
Clustering coefficient	0.328e-3	0.899e-3	1.07e-3
Global efficiency	0.451e-3	1.20e-3	1.38e-3
Diversity	18.2e-3	57.2e-3	55.5e-3
Modularity	10.5e-3	11.4e-3	9.78e-3

#### Intra-Class Correlation Coefficient

The computed ICC results and statistics are listed in **Table 3**, comparing different methods for six global network metrics. For all global metrics, the ICC of the mean of global network metrics from PBS is always higher than the mean ICC computed when taking each PBS sampling as one template. Five of the six global metrics show higher ICC from PBS than using the Shen atlas.

**Table 3:** Comparison of ICC of different methods for six global network metrics.

	ICC of mean	Mean ICC	*p*-Value	ICC template
Degree	0.533	0.508	1.3e-23	0.322
Strength	0.657	0.639	1.8e-30	0.656
Clustering coefficient	0.630	0.585	1.2e-58	0.571
Global efficiency	0.639	0.594	6.6e-59	0.630
Diversity	0.383	0.360	1.0e-29	0.464
Modularity	0.175	0.094	1.1e-30	-0.049

*ICC of mean is the ICC value when taking the mean value of global network metrics from PBS for each subject. Mean ICC is the average ICC values of the network metrics from individual parcellation. The *p*-value of the hypothesis that ICC of mean is greater than the ICC with a template of random parcellation was calculated from 400 trials. ICC template is the ICC value from the Shen atlas*.

### Fingerprint of Functional Network

**FIGURE 3:**
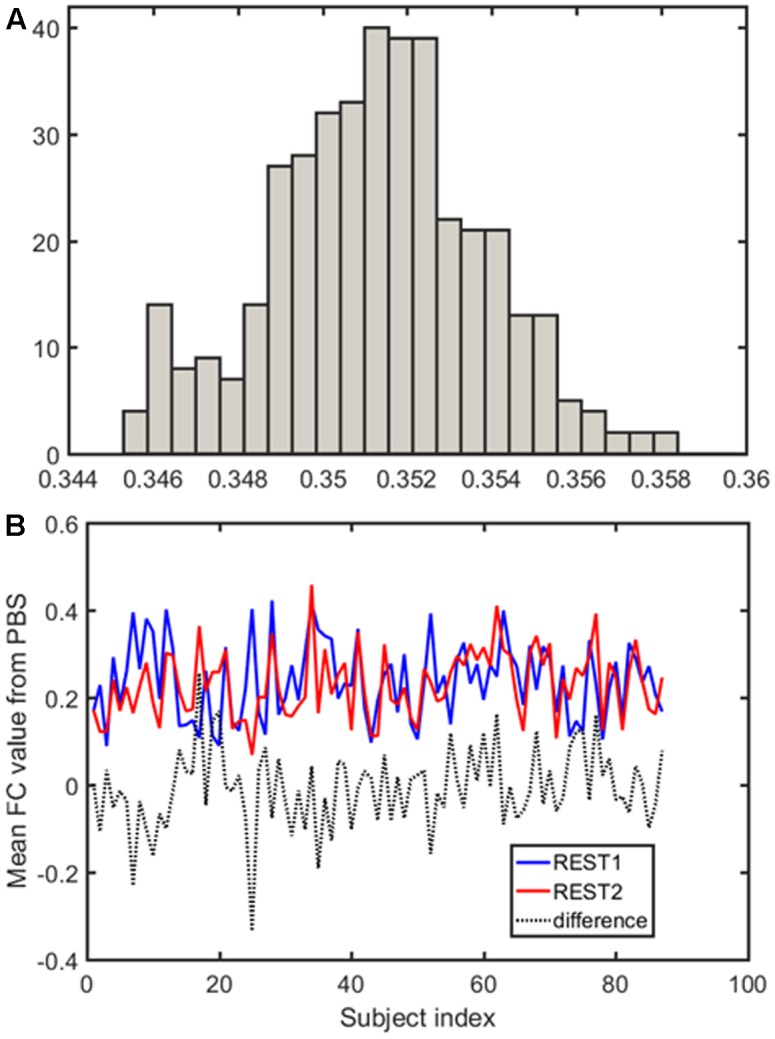
Each pseudo-bootstrap random parcellation gives rise to a FC matrix and a mean FC, an example of the distribution of the mean FC from 400 samples is shown in **(A)**. The mean FC values of all subjects in REST1 and REST2 along with their differences are plot in **(B)**.

**FIGURE 4:**
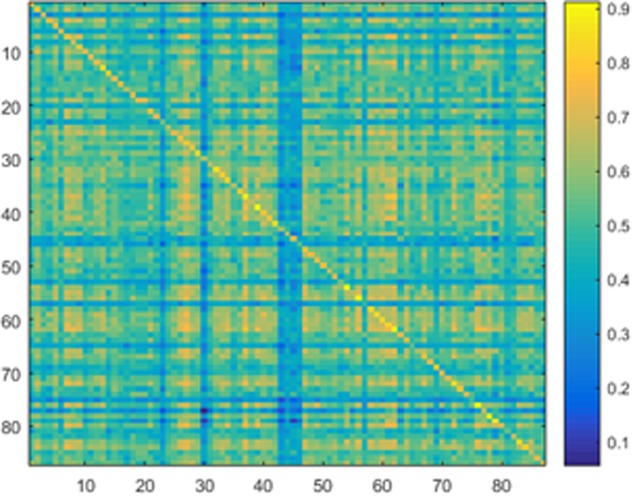
Correlation matrix of the inter-subject mean FC derived from the Pearson pair-wise cross-correlation between the vectors of 400 mean FC from Pseudo-Bootstrap samples. This correlation matrix represents the likelihood between subjects in terms of the coherence of change of mean FC with parcellation.

**FIGURE 5:**
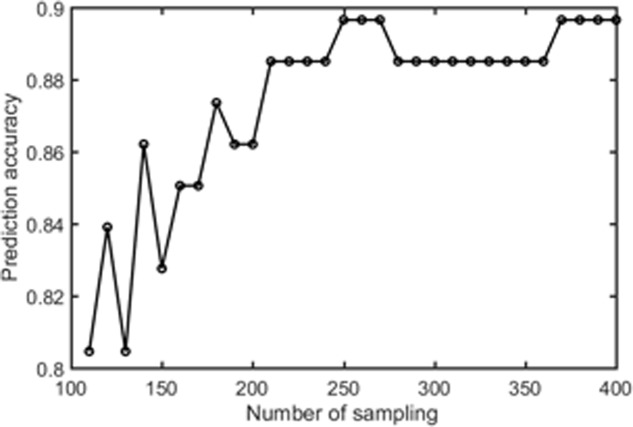
The prediction accuracy rate as a function of number of pseudo-bootstrap samples.

**FIGURE 6:**
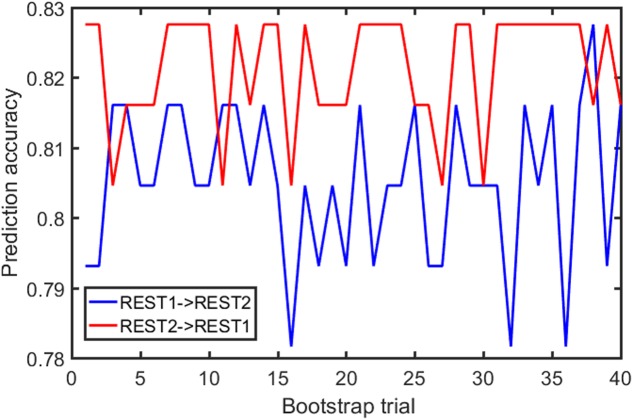
FC fingerprinting prediction accuracy rate based on the correlation of FC from template-based parcellation. The template was drawn from the PBS samples.

Samples of 400 PBS were obtained for each subject per resting session. Each PBS random parcellation generates a FC matrix and the corresponding mean FC, an example of the distribution of the mean FC from 400 PBS samples is shown in **Figure 3A**. The standard deviation of this distribution for all subjects is (2.05 ± 0.67) × 10^-3^ for REST1 and (1.89 ± 0.58) × 10^-3^ for REST2. The mean FC values of all subjects in REST1 and REST2 along with their differences are plotted in **Figure 3B**. **Figure 3B** shows that for some subjects, the mean FC value can be dramatically different between REST1 and REST2, compared with the mean standard deviation. **Figure 4** displays the correlation matrix of the inter-subject mFCV. This correlation matrix represents the likelihood between subjects in terms of the coherence of change of mean FC with parcellation. By searching for the maximum value corresponding to row index or column index, prediction accuracy is 0.885 from 1 to 2 and 0.897 from 2 to 1. The prediction accuracy is a function of the sampling number as shown in **Figure 5**. As the number of samples is decreased from 400 to 110, the accuracy drops to around 0.8. This accuracy is comparable to the method directly comparing network matrix from a single parcellation, as shown in **Figure 6**. Using the Shen atlas, the prediction rate is 82.6% from REST2 to REST1 and 83.7% from REST1 to REST2.

## Discussion

A new framework for network analysis is proposed based on PBS sampling, implemented as the generation of multiple random parcellations on a single MRI volume. Because small parcel-size variation across different samples (instances of a random parcellation) is critical to ensure comparable network metrics across several parcellation trials, we propose a random parcellation algorithm that can produce sets of random parcellations with a given number of parcels with a small parcel-size variability. The inter-quartile range to median ratio is around 0.10, significantly smaller than previous results: 0.77 (Smith et al., 2004) and 0.52 (Echtermeyer et al., 2011). Another advantage of this algorithm is that it generates the number of nodes exactly as specified.

The impact of PBS on structural network highlights a lot of information about the effect of parcellation on network metrics. The PBS sampling resulted in a Gaussian-like distribution of the global metrics, indicating that different parcellation can lead to similar global metric values. Nonetheless, it is worth noting that such values do vary. On the other hand, while the global network properties of the structural brain network vary across different repetitions of the equally sized random parcellations, we find that the variability is small and comparable with inter-subject variability and within-subject variations between dMRI scans. Our results show that parcellation is only one source to variation of network properties. It is more critical to reduce variances from measurements, fiber tracking, etc. The ICC shows that the mean value of global metrics from PBS tests is larger than the mean value of template-based parcellation. Although it does not give the best ICC in general, it is better than a template-based parcellation overall. Given the lack of a standard parcellation scheme, the PBS sampling with random parcellation may be a plausible method to perform network analysis.

Our results show that a higher prediction accuracy rate was achieved for FC fingerprint with pseudo bootstrap parcellation compared to template-based parcellation. The prediction rate, as expected, is dependent on the number of samples. The results suggest that random parcellation analysis opens a new window to examine functional networks, which preserve some features that are insensitive to resampling at certain scale. Each resampling can be considered a coarse snapshot of the true FC network from different angles. The coherences between snapshots is a unique feature of FC fingerprinting that has never been explored before. Only the mean FC strength was tested in this article, it does not exclude other global network properties that are preserved as well.

The PBS sampling with random parcellations is different from the template-based parcellation. In fact, the parcels have neither functional nor anatomical meanings but this lack of meaning can be an advantage in that there is no risk of introducing false assumptions or biases into the network model. On the other hand, a template-based parcellation does not belong to the set of PBS samples in general because the criteria to generate the parcellation is completely different. An advantage of the PBS approach is bringing rich statistical analysis on the networks that addresses the variation of global network properties related to parcellation. For instance, the distribution of global metrics might be different for different subjects but similar for the same subject. Unfortunately, the sample size was not high enough to run any of the statistics effectively. Another advantage of PBS over template-based parcellation is reducing the inter-subject variability due to parcellation when comparing different subjects. This can be clearly revealed by the fact that ICC of the mean is higher than the mean ICC for all global networks and the *p*-values of the tailed *t*-test that ICC from PBS using the mean is higher than that from template-based parcellation using one random parcellation are extremely low (**Table 3**).

Like most parcellation schemes, the random parcellation algorithm proposed in this paper works in the 3D volume space. A surface-based random parcellation has been proposed previously in an attempt to build an atlas-free framework for constructing and comparing connectomes (Tymofiyeva et al., 2014). While that framework shares the same goal as ours, a challenge of that method is the mandatory network alignment prior to comparing connectomes. Network alignment is a procedure to minimize the “distance” between networks by reordering nodes. For small networks the alignment can be achieved by simply permuting the nodes. However, this approach is not practical when the size of network gets large because the number of permutations is the factorial of the number of nodes. The quality of alignment is subject to the algorithm and computation time. In fact, the PBS concept can be readily combined with the surface-based random parcellation on individual level and there is no need to align the network for comparing global network metrics.

One undesirable feature of the PBS methods is that the computation time can be long. For individual subjects, the computation time is a multiplication of the time to generate each random parcellation and subsequent network construction/analysis by the number of PBS samples. For group analysis, one can use predefined random parcellations and the multiple sampling only adds time in subsequent calculations. In any case, the computation time is much longer than template-based methods. However, with advancements in high-throughput computing clusters and high-performance parallel computing, it becomes less a problem in real application. In addition, more work is needed for the optimization of PBS method. For instance, what is he optimal parcel size? Because this method completely ignores functional and anatomical information of the image data, big parcels are usually not good representatives of network nodes; but very small parcel size leads to less variability (an extreme case is voxel-wise parcellation (Power et al., 2011)) of the network. Moreover, the PBS sampling only varies in brain parcellation. As shown from our data, the variation of the network metrics could be affected more by other factors than random parcellations. Hence, more sophisticated statistical methods are desired to extract insights of intrinsic brain network properties from the variation of the network metrics. The fingerprinting using the PBS and correlation of mean FC is one example of making use of the variations.

## Conclusion

In summary, a new algorithm was proposed to obtain roughly equal-sized random parcellations by considering the geodesic distance between voxels and voxel density. By applying these random parcellations to individual subjects multiple times, a PBS of the network was obtained. One benefit of PBS network analysis over conventional approaches based on template-based parcellations is the higher ICC of global network metrics. An application of PBS sampling on FC fingerprinting showed higher accuracy than previous method using the correlation of the FC matrices. While a golden rule for choosing brain network nodes remains lacking, the results from our preliminary work encourages a more thorough understanding of the statistical nature of this method.

## Ethics Statement

The human data used in this study are from studies approved by the Internal Review Board of Indiana University School of Medicine.

## Author Contributions

HC involved in all aspects of the work and wrote the manuscript. AL and CH worked on the FC fingerprinting and statistical inferences. AK worked on the random parcellation algorithm and helped with manuscript editing. YW and JS worked on the structural network analysis. SN involved in all aspects of the work and helped with manuscript editing.

## Conflict of Interest Statement

The authors declare that the research was conducted in the absence of any commercial or financial relationships that could be construed as a potential conflict of interest.

## Footnotes

1 http://trackvis.org/
